# Supplementary material for: Dietary Sources of Salt in Low- and Middle-Income Countries: A Systematic Literature Review
Source: Int J Environ Res Public Health. 2019 Jun 12;16(12):2082. doi: 10.3390/ijerph16122082 (PMC6617282; doi:10.3390/ijerph16122082)
Supplement: Supplementary file 1 [file ijerph-16-02082-s001.pdf]

Table S1. Search strategy.

Searches	Search Terms
Medline Scopus Web of Science	"source of salt" OR "dietary salt" OR "discretionary salt" OR "salt intake" OR "salt in food" OR "salt added at the table" OR "dietary sources of sodium" **AND** "Algeria" OR "Angola" OR "Benin" OR "Botswana" OR "Burkina Faso" OR "Burundi" OR "Cameroon" OR "Cape Verde" OR "Central African Republic" OR "Chad" OR "Democratic Republic of Congo" OR "Republic of Congo" OR "Cote d'Ivoire" OR "Equatorial Guinea" OR "Ethiopia" OR "Gabon" OR "Gambia" OR "Ghana" OR "Guinea" OR "Guinea Bissau" OR "Kenya" OR "Lesotho" OR "Liberia" OR "Madagascar" OR "Malawi" OR "Mauritius" OR "Mozambique" OR "Namibia" OR "Nigeria" OR "Reunion" OR "Rwanda" OR "Sao Tome and Principe" OR "Senegal" OR "Seychelles" OR "Sierra Leone" OR "South Africa" OR "Swaziland" OR "Tanzania" OR "Togo" OR "Uganda" OR "Zambia" OR "Zimbabwe" OR "Afghanistan" OR "Albania" OR "American Samoa" OR "Armenia" OR "Azerbaijan" OR "Bangladesh" OR "Belarus" OR "Belize" OR "Bhutan" OR "Bolivia" OR "Bosnia and Herzegovina" OR "Brazil" OR "Bulgaria" OR "Cambodia" OR "China" OR "Colombia" OR "Comoros" OR "Costa Rica" OR "Cuba" OR "Macedonia" OR "Malaysia" OR "Maldives" OR "Marshall Islands" OR "Mexico" OR "Micronesia, Fed. Sts." OR "Moldova" OR "Mongolia" OR "Montenegro" OR "Myanmar" OR "Nepal" OR "Nicaragua" OR "Pakistan" OR "Palau" OR "Panama" OR "Papua New Guinea" OR "Paraguay" OR "Peru" OR "Philippines" OR "Romania" OR "Rwanda" OR "Samoa" OR "Dominica" OR "Dominican Republic" OR "Ecuador " OR "Serbia" OR "El Salvador" OR "Solomon Islands" OR "Fiji" OR "Sri Lanka" OR "St. Lucia" OR "Georgia" OR "St. Vincent and the Grenadines" OR "Grenada" OR "Suriname" OR "Guatemala" OR "Syria" OR "Tajikistan" OR "Guyana" OR "Haiti" OR "Thailand" OR "Honduras" OR "Timor-Leste" OR "India" OR "Indonesia" OR "Tonga" OR "Iran" OR "Iraq" OR "Turkey" OR "Jamaica" OR "Turkmenistan" OR "Jordan" OR "Tuvalu" OR "Kazakhstan" OR "Ukraine" OR "Kiribati" OR "Uzbekistan" OR "Korea, Dem. People's Rep." OR "Vanuatu" OR "Kosovo" OR "Vietnam" OR "Kyrgyz Republic" OR "West Bank and Gaza" OR "Lao PDR" OR "Yemen" OR "Lebanon"

Table S2. Risk of bias assessment.

Included Studies	Were the Aims/Objectives of the Study Clear?	Was the Design Appropriate for the Aim(s) Stated?	Was the Sample Size Justified?	Was the Target Population Clearly Defined?	Was the Sample Taken From an Appropriate Population Base?	Was the Selection Process Able to Select a Representative Sample?	Were Measures Undertaken to Address And Categorize Non-Respondents?	Were Risk Factors and Outcome Measured Appropriate to the Aims of the Study	Is it Clear What Was Used to Determine Statistical or Precision Estimates?
Kerry et al., 2005	Yes	Yes	No	Yes	Yes	Yes	Yes	Yes	Yes
Ferrante et al., 2011	Yes	Yes	No	Yes	Yes	No	Yes	Yes	Yes
Charlton et al., 2005	Yes	Yes	No	Yes	Yes	No	No	Yes	Yes
Liu et al., 2014	Yes	Yes	No	Yes	Yes	No	Yes	Yes	Yes
Zhao et al., 2015	Yes	Yes	No	Yes	Yes	No	No	Yes	Yes
Health Promotion Board, Singapore, 2011	Yes	Yes	No	Yes	Yes	Yes	Yes	Yes	Yes
Du et al., 2014	Yes	Yes	No	Yes	Yes	Yes	Yes	Yes	Yes
de Moura Souza, 2013	Yes	Yes	No	Yes	Yes	Yes	Yes	Yes	Yes
Anderson et al., 2010	Yes	Yes	No	Yes	Yes	No	Yes	Yes	Yes

Table S3. Included articles and their regions of origin.

Reference	Country	Data Collection Period	Study Emphasis	Study Design
**Americas**				
Ferrante et al., 2011 [40]	Argentina	2006 and 2007	Product Reformation	Descriptive
De Moura Souza et al., 2013 [48]	Brazil	2008–2009	Dietary Behaviour	Descriptive
**Western Pacific**				
Liu et al., 2014 [51]	China	April to Dec, 2011	Dietary Behaviour	Descriptive
Du et al., 2014 [53]	China	1991 to 2009	Dietary Behaviour	Descriptive
Anderson et al., 2010 [55]	China	1996–1999	Dietary Behaviour	Descriptive
Zhao et al., 2015 [49]	China	2011	Dietary Behaviour	Descriptive
**South East Asia**				
Khan et al., 2011 [54]	Bangladesh	October 2009 through March 2010	Dietary Behaviour	Descriptive
Health Promotion Board, 2011 [52]	Singapore	Not reported	Dietary Behaviour	Descriptive
**Eastern Mediterranean**				
Hussain & Takruri, 2016 [42]	Jordan	Not reported	Product Reformation	Descriptive
Zibaeenezhad et al., 2010 [43]	Iran	Not reported	Product Reformation	Descriptive
**Europe**				
Vukic et al., 2013 [45]	Bosnia Herzegovina	Not reported	Product Reformation	Descriptive
**Africa**				
Silva et al., 2015 [41]	Mozambique	June, 2012	Product Reformation	Descriptive (Cross sectional)
Charlton et al., 2005 [50]	South Africa	Not reported	Dietary Behaviour	Descriptive (Cross sectional)
Kerry, 2005 [58]	Ghana	June 2001 and June 2002	Dietary Behaviour	Cluster randomized, controlled trial
Nwanguma, 2013 [36]	Nigeria	Not reported	Product Reformation	Descriptive (Cross sectional)
